# Impact of *TP53* loss-of-function alterations on the response to PSMA radioligand therapy in metastatic castration-resistant prostate cancer patients

**DOI:** 10.7150/thno.96322

**Published:** 2024-08-01

**Authors:** Peter H.J. Slootbeek, María Victoria Luna-Velez, Bastiaan M. Privé, Maarten J. van der Doelen, Iris S.H. Kloots, Samhita Pamidimarri Naga, Hilde E. Onstenk, James Nagarajah, Harm Westdorp, Inge M. van Oort, Leonie I. Kroeze, Jack. A. Schalken, Haiko J. Bloemendal, Niven Mehra

**Affiliations:** 1 Department of Medical Oncology, Radboud university medical center, Nijmegen, The Netherlands.; 2 Department of Urology, Radboud university medical center, Nijmegen, The Netherlands.; 3 Department of Nuclear Medicine, Radboud university medical center, Nijmegen, The Netherlands.; 4 Roentgeninstitut Duesseldorf, Duesseldorf, Germany.; 5 Department of Pathology, Radboud university medical center, Nijmegen, The Netherlands.

**Keywords:** Castration-resistant prostate cancer, Precision oncology, *TP53*, Theranostics, Radioligand therapy

## Abstract

**Rationale:** PSMA-targeting radioligand therapy (PSMA-RLT) has shown promise in metastatic castration-resistant prostate cancer (mCRPC), particularly in PSMA-avid tumours. However, predicting response remains challenging. Preclinical data suggests aberrant p53-signalling as a predictor of poor response.

**Methods:** The patient population of this pre-planned retrospective cohort study consists of 96 patients with mCRPC who underwent treatment with PSMA-RLT and were molecularly profiled by whole-genome sequencing and or targeted next-generation sequencing. Response to PSMA-RLT was assessed per molecular subtype, including *TP53*-mutational status.

**Results:** Patients with* TP53* loss-of-function alterations had a shorter median progression-free survival (3.7 versus 6.2 months, *P*<0.001), a lower median PSA change (-55% vs. -75%, *P*=0.012) and shorter overall survival from initiation of PMSA-RLT (7.6 vs. 13.9 months, *P*=0.003) compared to *TP53*-wildtype patients. Pathogenic alterations in *AR*, *MYC*, *BRCA1*, or *BRCA2* as well as in genes linked to the PI3K or MAPK pathways or genes involved in homologous recombination repair, were not associated with response. Only lactate dehydrogenase was, alongside *TP53*-status, significantly associated with response. Transcriptome analysis of 21 patients, identified six p53 signalling genes whose low expression was associated to a shorter progression-free survival (*P*<0.05).

**Conclusion:** *TP53* loss-of-function may serve as a prognostic factor for PSMA-RLT outcomes in patients with mCRPC.

## Introduction

Although the armamentarium for metastatic castration-resistant prostate cancer (mCRPC) has substantially expanded over the last decade, patients almost inevitably progress on all registered treatment lines, resulting in a median life expectancy of less than three years 1-3. In the search to further broaden the treatment options of mCRPC patients, radioligand therapy (RLT) has gained momentum. The most common cell-surface protein used to guide radiopharmaceuticals towards prostate cancer cells is the prostate specific membrane antigen (PSMA) 4, 5. As PSMA is overexpressed in prostate cancer cells compared to benign tissue, the therapeutical radiation dose is accumulated at the tumour site, limiting radiation damage to non-PSMA-expressing tissues and reducing damage to healthy tissues.

PSMA ligands can be labelled with radioisotopes such as the beta-emitter lutetium-177 (^177^Lu) or the alpha-emitter actinium-225 (^225^Ac) 6, 7. The VISION trial led to the EMA and FDA approval of ^177^Lu-PSMA post-taxane, based on improved progression-free survival (PFS) and overall survival (OS) and while the final results of the PSMAfore study are pending, approval of ^177^Lu-PSMA for taxane-naive patients is anticipated, as the trial presented a significantly prolonged PFS 8, 9. ^225^Ac-PSMA has not reached the phase 3 trial stage, but several phase 2 trials are currently ongoing (NCT03276572, NCT04506567, NCT05219500, NCT04597411). Tandem therapy with ^177^Lu-PSMA and ^225^Ac-PSMA has shown promising results, even after progression on single-agent ^177^Lu-PSMA. This is currently investigated in a phase 2 trial (NCT04886986) 10, 11.

For newly approved therapies in an all-comer population, such as PSMA-RLT, an unmet need is the identification of biomarkers that guide physicians to select responsive patients more optimally. As PSMA avidity strongly influences response, the landmark papers of LuPSMA, TheraP and VISION excluded patients with PSMA low or negative lesions based on relative uptake compared to the liver, a threshold maximum standardised uptake value (SUVmax) per lesion or mismatch with FDG-PET 8, 12-14. Yet, post-hoc analyses of the VISION and TheraP showed that there are still many responders with intermediate PSMA uptake 15, 16. Hence, exploring additional biomarkers is warranted.

Preclinical evidence supports p53 (encoded by *TP53*) signalling as an important biomarker candidate. In a study by Stuparu *et al.*, global proteomics and phosphoproteomics were used to investigate the molecular changes induced by PSMA-RLT in mice 17. Transcription factor enrichment analysis revealed that p53 was the most upregulated transcription factor post ^177^Lu-PSMA RLT and the third most upregulated post ^225^Ac-PSMA RLT. Additionally, kinase-substrate enrichment analysis showed increased activity of ATM and ATR in mice treated with RLT, and increased activity of CHK2 (encoded by *Chek2*) in ^177^Lu-PSMA treated mice. Interestingly, ATM, ATR and CHK2 are all involved in the stabilization and activation of p53 in response to ionizing radiation 18. To further confirm these findings, the authors assessed the impact of *Tp53* status on PSMA-RLT responsiveness in mice. They found that PSMA-RLT was effective in mice with wild-type *Tp53* tumours but much less in mice with *Tp53* knock-out tumours, with no significant reduction in tumour growth compared to untreated mice. From literature evaluating the genetic background of mCRPC patients treated with PSMA-RLT, *TP53* status could not be evidently validated as a biomarker associated with response 19-21.

In this pre-planned retrospective cohort study, we hypothesised that mCRPC patients with loss-of-function alterations in *TP53* would respond worse to PSMA-RLT when compared to patients with wild-type *TP53*. To test this hypothesis, we performed a comprehensive molecular characterization of 96 patients with mCRPC and evaluated the response to PSMA-RLT per molecular subtype, including TP53-mutational status. Lastly, transcriptome analysis was performed to identify signalling pathways and constituent genes associated to biochemical progression and the loss of p53 signalling.

## Methods

### Patient population and study design

The patient population of this pre-defined retrospective cohort study consisted of all patients known in the outpatient clinics of Medical Oncology or Nuclear Medicine at the Radboudumc, treated with ^177^Lu-PSMA or ^225^Ac-PSMA, from January 1, 2016, to May 1, 2023. Follow-up data were collected until November 1, 2023. Eligible patients previously underwent next-generation sequencing of tumour tissue (fresh or archived) or had residual tumour tissue from earlier biopsies. Different consents were allowed to be included in this study, all specified in study protocol, evaluated by the Medical Review Ethics Committee Oost-Nederland, The Netherlands (CMO-2022-16040). The study population in part overlaps with the study populations of previous publications from our centre with different research questions 22, 23.

The pre-planned primary research objective was to compare PFS on PSMA-RLT between patients with pathogenic *TP53* alterations and patients without pathogenic *TP53* alterations. The secondary endpoints were PSA response and overall survival per *TP53* status. Patients were classified as *TP53* mutated (TP53m) if they had a bi-allelic loss of *TP53*, a relevant splice-site mutation, a mutation in *TP53* with a truncating effect or a missense mutation with a non-functional transcriptional activity according to The *TP53* Database (R20, July 2019): https://tp53.isb-cgc.org 24. Patients with non-deleterious alterations in *TP53* or mutations with a partially functional transcriptional activity were included in the *TP53* wild-type (TP53wt) subgroup.

PFS on PSMA-RLT was defined as the time from first administration of PSMA-RLT until radiologic or clinical progression including death or censoring at end of follow-up if treatment was still ongoing. PSA responses were assessed as maximal decline according to the Prostate Cancer Clinical Trials Working Group (PCWG3) criteria and dichotomised by ≥50% PSA decline (PSA50) 25. Biochemical PFS was defined as the time from first administration of PSMA-RLT until ≥25% PSA increase from the nadir or baseline if PSA did not decline, censoring at next-systemic therapy, end of follow-up or death.

### Molecular analysis

All patients underwent targeted or whole-genome sequencing (WGS) on primary or metastatic tissue by a non-profit institute (Hartwig Medical Foundation; WGS), by a fee for service provider (Foundation Medicine; Foundation One CDx) and/or in-house using a commercially available targeted sequencing panel containing 523 cancer-related genes (Illumina; True Sight Oncology 500) 26. To compare the relative impact of *TP53* loss-of-function alterations to presumed hyperactivation of canonical oncogenic pathways (AR, PI3K, MAPK, MYC) or impairment of homologous recombination repair (HRRm), all patients were sequenced for at least the following genes: *TP53, AR, RB1, PTEN*, *AKT1*, *AKT2*, *AKT3*, *PIK3CA*, *PIK3CB*, *PIK3R1*, *BRAF*, *MAP2K1*, *MAP2K2*, *MAP2K4*, *MAP3K1*, *MYC*, *ATM, BARD1, BRCA1*, *BRCA2, BRIP1, CDK12, CHEK1, CHEK2, FANCA, FANCL, NBN, PALB2, PPP2R2A, RAD51B, RAD51C, RAD51D, RAD54L*. To ensure consistency in pathogenicity reporting, all external sequencing reports were re-assessed based on guidelines from the American College of Medical Genetics and Genomics and the Association for Molecular Pathology 27, 28. Genes with six copies or more according to the reporting service or calculated inhouse were considered amplified 26. Genes with no copies were reported as loss.

### Transcriptome analysis

The transcriptional activity of p53 was explored with a gene-set enrichment analysis (GSEA). DESeq2 (v1.38.3) was used to normalise and log2-transform the raw count data from RNA sequencing of 21 mCRPC patients treated with PSMA-RLT 29. For the enrichment analysis, patients were divided into groups to calculate fold change transcript expression between patients with and without a PSA50 and between TP53m and TP53wt. Log2 fold change values were calculated with the R package apeglm (v1.14.0) with the adaptive shrinkage estimator “ashr”, and used as input for the GSEA 30. GSEA was performed using the molecular signatures database (MSigDB) hallmark gene set collection (v7.5.1) with the fgsea R package (v1.27.0) 31, 32. Expression heatmaps of the normalised, log2-transformed data were created with ComplexHeatmap (v2.10.0) 33.

### Statistical analyses

Time-to-event data were compared using Cox proportionate hazard models and visualised with Kaplan-Meier curves. Multivariable Cox proportionate hazard models were used to assess the impact of different molecular subgroups simultaneously and to test the impact of TP53m status on response relative to the line of therapy, time from androgen deprivation to mCRPC, type of isotope used, and the baseline laboratory values: prostate specific antigen (PSA), lactate dehydrogenase (LDH), haemoglobin (HB), alkaline phosphate (ALP). The proportional hazards assumption was tested with the Schoenfeld Test. To investigate the impact of single genes within the *TP53* signature from the GSEA on PFS, the median value of the normalised and log2-transformed expression of each gene was used to separate patients into two groups, 50% highest and 50% lowest expression, which were compared using a log-rank test. To compare the baseline characteristics and biochemical outcomes of the subgroups, categorical variables were analysed using the Pearson Chi-Square or Fisher's Exact Test. Continuous variables were assessed using the nonparametric Mann-Whitney U test. All statistical tests were two-sided, with *P* values <0.05 considered statistically significant. All statistical tests and data visualization were performed in R (version 4.1.3) with RStudio (version 2022.02.1). A statistician was consulted during the analyses.

## Results

### Patient cohort

**Figure 1:**
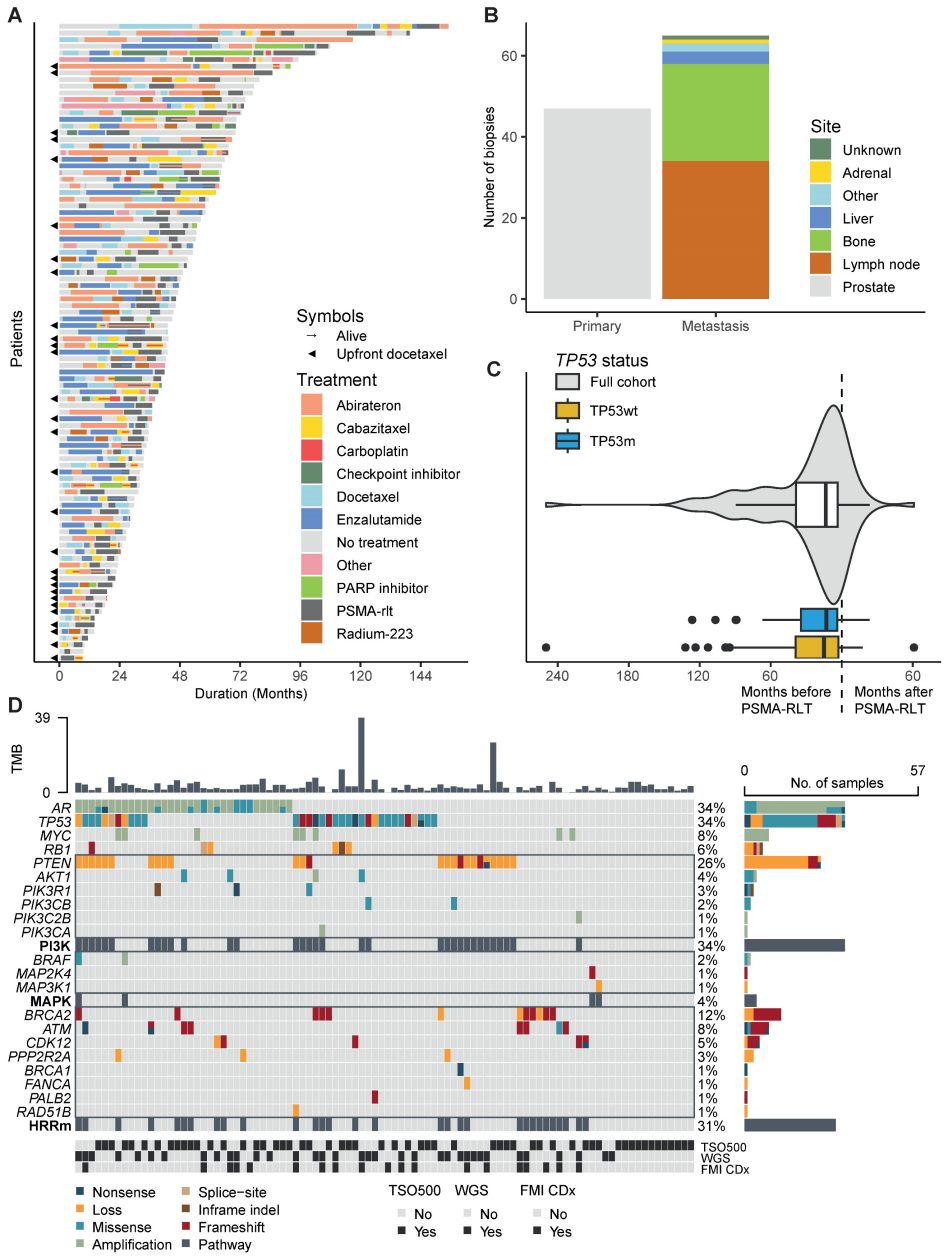
** A.** Swimmerplot presenting the order and duration of systemic life-prolonging therapies for castration-resistant prostate cancer until death or last follow-up. The colour scheme represents therapies, and the symbols indicate if patients received upfront docetaxel or were alive at last follow-up. **B**. Barchart showing the sites from which biopsies were taken. **C**. Violin- and boxplots showing the timing of biopsies relative to the initiation of PSMA-RLT. **D.** Oncoplot presenting the genetic aberrations. The colour of the boxes represents the effect of the alteration, sorted by pathway. The tumour mutational burden (TMB) is presented at the top and at the bottom the different sequencing methods are presented. Abbreviations: FMI CDx, FoundationOne® companion diagnostic; HRRm, homologous recombination repair mutated (including loss); PSMA-RLT, prostate-specific membrane antigen-targeting radioligand therapy; TSO500, TruSight Oncology 500; WGS, whole genome sequencing.

In total, 96 mCRPC patients were included in the study population. Patients were treated with a median of four systemic treatment lines for mCRPC before start of PSMA-RLT. The complete therapy sequence from mCRPC until last follow-up for each patient is presented in Figure 1A. Among the 96 patients, we analysed 112 tumour samples. Almost half of the samples were prostate tissue (42%), 30% were tissue from lymph nodes and 21% from bone (Figure 1B). The median time between obtaining the tissue and initiation of PSMA-RLT was 13.5 months (interquartile range 3.4 - 38.6, Figure 1C). Thirteen biopsies were taken after initiation of PSMA-RLT. The most frequently pathogenically altered genes were *AR* (34%) and *TP53* (34%), followed by *PTEN* (26%) and *BRCA2* (12%) (Figure 1D). Baseline characteristics did not significantly differ between the 33 patients (34%) in the TP53m subgroups and 63 patients (66%) in the TP53wt subgroup (Table 1). Sixty-seven patients received ^177^Lu-PSMA as single-agent, seven patients received ^225^Ac-PSMA as single-agent, and thirteen patients received tandem therapy with ^177^Lu-PSMA and ^225^Ac-PSMA.

TP53m patients received less cycles of PSMA-RLT compared to TP53wt patients (median 3 versus 4 cycles, *P*=0.028; Supplementary Table 1). Only one in four TP53m patients received four or more cycles (Table 1). In total, 89 patients received ^177^Lu-PSMA with a median total activity of 22.2GBq; 16.5GBq for the TP53m subgroups and 24.0GBq for the TP53wt subgroup (*P*=0.110). The median total activity for the 20 patients receiving ^225^Ac-PSMA was 14.3MBq; 8.0MBq for the TP53m patients and 20.0MBq the TP53wt patients (*P*=0.025).

### Progression-free survival

**Figure 2:**
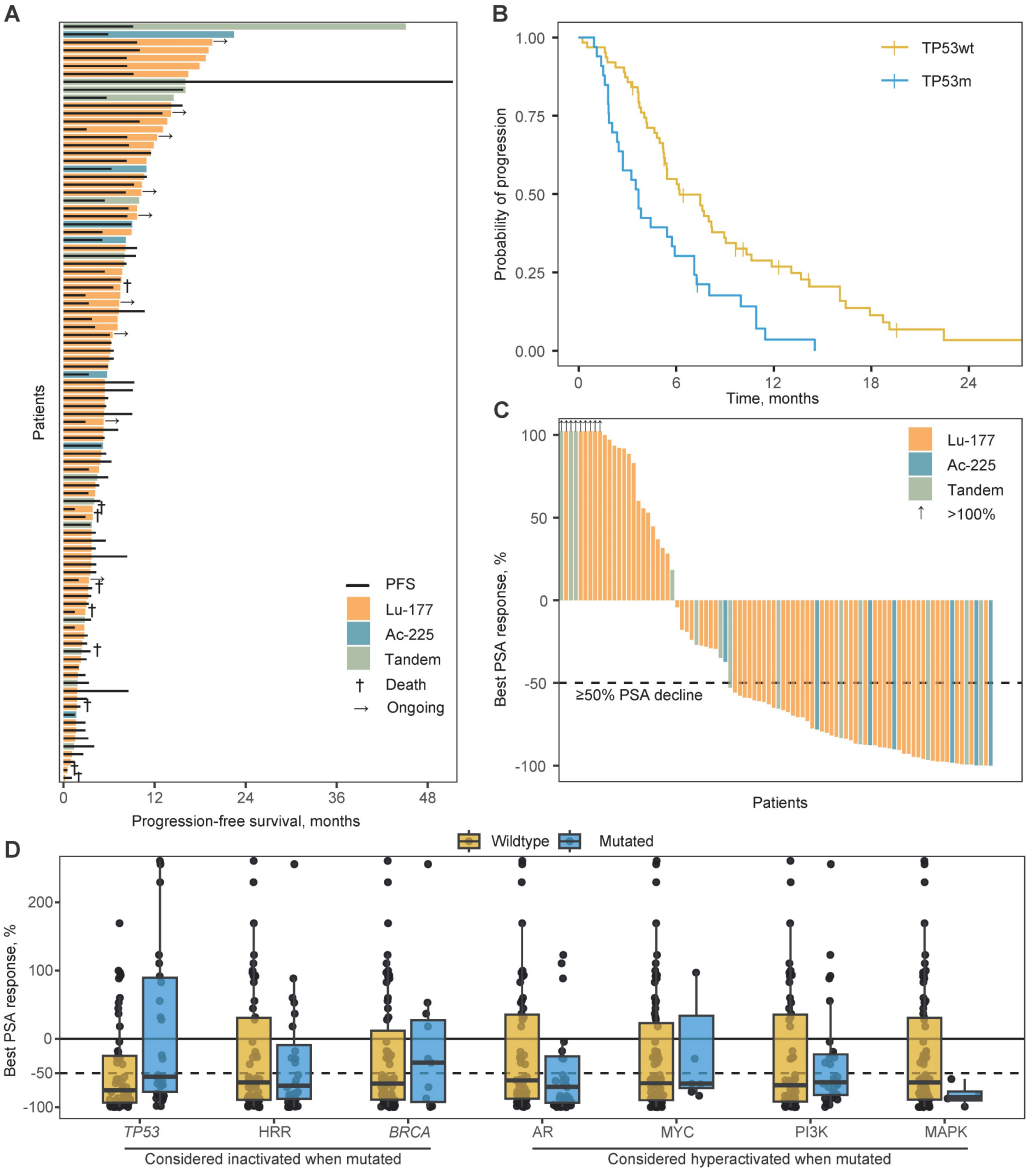
** A.** Swimmerplot presenting time on treatment per type of radioligand by coloured bars. The black lines indicate radiographic or clinical progression-free survival. **B.** Kaplan-Meijer curves for the progression-free survival per *TP53*-mutational status. **C.** Waterfallplot presenting the best prostate specific antigen (PSA) response from baseline per type of radioligand. **D.** Boxplot with individual points presenting the best PSA response per mutational status for canonical oncogenic or tumour suppressive pathways. Per boxplot: center line, median; box limits, upper and lower quartiles; from box to largest and smallest point within box + 1.5x interquartile range. Abbreviations: Ac-225, actinium-225; HHR, homologous recombination repair; Lu-177, lutetium-177; PSA, prostate specific antigen.

The median PFS on PSMA-RLT for the total population was 5.4 months (95% confidence interval [CI] 4.8 - 7.5) and was not impacted by type of RLT (*P*=0.432; Figure 2A). The TP53m subgroup had a significantly shorter PFS when compared to the TP53wt subgroup (median 3.7 versus 6.2 months; hazard ratio [HR] 2.2, 95%CI 1.4 - 3.5; *P*<0.001; Figure 2B). The hazard ratio for ^177^Lu-treated patients was 2.3 (95%CI 1.4 - 3.7; *P*<0.001) and for ^255^Ac-treated patients 2.0 (95%CI 0.7 - 5.4; *P*=0.177). HRRm, deleterious alterations in *BRCA1* or *BRCA2* specifically (BRCAm), as well as activating alterations in *AR*, *MYC* or key genes in the PI3K and MAPK pathway, were univariably not associated with PFS on PSMA-RLT (Table 2). In a multivariable analysis, only *TP53*-status was significantly associated with PFS (Table 2). Even when corrected for prognostic factors or possible confounders, *TP53* status remained significantly associated with PFS (*P*=0.005). Only baseline LDH level was also significantly associated (*P*=0.001) with PFS (Supplementary Table 2). Notably, SUVmax did not show a significant association with PFS (*P*=0.703).

#### Exploratory analyses for progression-free survival

Patients with a molecular signature of aggressive variant prostate cancer (AVPC, n = 12), comprised of loss-of-function alterations in at least two of the three genes: *TP53*, *RB1*, *PTEN*, had a shorter PFS on PSMA-RLT (HR 1.8; 95%CI 1.0 - 3.4; Supplementary Figure 1). However, with a lower hazard ratio as *TP53*-status alone, suggesting *TP53* loss-of-function drives the poor response on PSMA-RLT in AVPC patients, especially since all AVPC patients were also TP53m. By combining *TP53*-status with loss-of-function alterations in the genes encoding for the key activators and stabilisers of p53 (*ATM*, *CHEK1*, and *CHEK2*), an additional 11 patients were considered as having impaired p53 signalling: seven patients due to alterations in *ATM* and four due to alterations in *CHEK2*. The 44 patients with impaired p53 signalling generally had a shorter PFS (HR 1.7; 95%CI 1.1 - 2.5; Supplementary Figure 1). This effect was not as pronounced as when the subgroups were formed based on *TP53*-status alone, suggesting that *TP53* is the main driver of a shorter PFS.

Although HRRm, or specifically BRCAm, was not associated with PFS in the full study population, HRRm might still be associated with PFS in patients treated with ^225^Ac-PSMA. ^225^Ac emits alpha-radiation, which is much more potent in inflicting double-stranded DNA breaks that are reliant on homologous recombination for error-free restoration when compared to beta-radiation. However, an exploratory analysis with only the 20 patients who received ^225^Ac-PSMA did not show an association between HRRm (n = 5) or BRCAm-status (n = 4) and PFS on ^225^Ac-PSMA (HRRm: HR 1.4; 95%CI 0.5 - 3.9; BRCAm: HR 1.2 95%CI 0.4 - 3.7).

### Biochemical response

The median PSA change for the total cohort was -65% (interquartile range -0.89 - 0.26) with 61% of patients having a PSA50 (Figure 2C). The median PSA change was significantly more beneficial for TP53wt patients when compared to TP53m patients (-75% vs. -55%; *P*=0.012; Figure 2D). The proportion of patients with a PSA50 did not significantly differ (65% vs. 53%, respectively; *P*=0.400). At 12 weeks after initiation of PSMA-RLT, the median PSA change was -56% for TP53wt patients and -36% for TP53m patients (*P*=0.064). A PSA50 was witnessed by 56% versus 45% of patients, respectively (*P*=0.451). Notably, evaluation at 12 weeks was hampered due to missing PSA values for 24 of the 96 patients (25%). None of the other genetic subgroups was statistically significant associated with either median PSA response or PSA50 (Figure 2D; Supplementary Table 3). Notably, all four patients with presumed hyperactivation of the MAPK pathway, did reach a PSA50.

#### Biochemical progression-free survival (exploratory)

The median biochemical PFS (bPFS) of the total population was with 4.3 months (95%CI 4.0 - 5.9 months) approximately one month shorter than the radiologic/clinical PFS. The bPFS for the TP53m subgroup was shorter when compared to the TP53wt subgroup (3.1 vs. 5.5 months; HR 1.8; 95%CI 1.1 - 2.8; Supplementary Figure 2) and remained significant when corrected for PSA at initiation of PSMA-RLT (HR 1.7; 95%CI 1.1 - 2.8). In a multivariable analysis, TP53m was the sole molecular subgroup significantly associated with bPFS (HR 2.5; 95%CI 1.5 - 4.2; Supplementary Table 4).

### Overall survival

**Figure 3:**
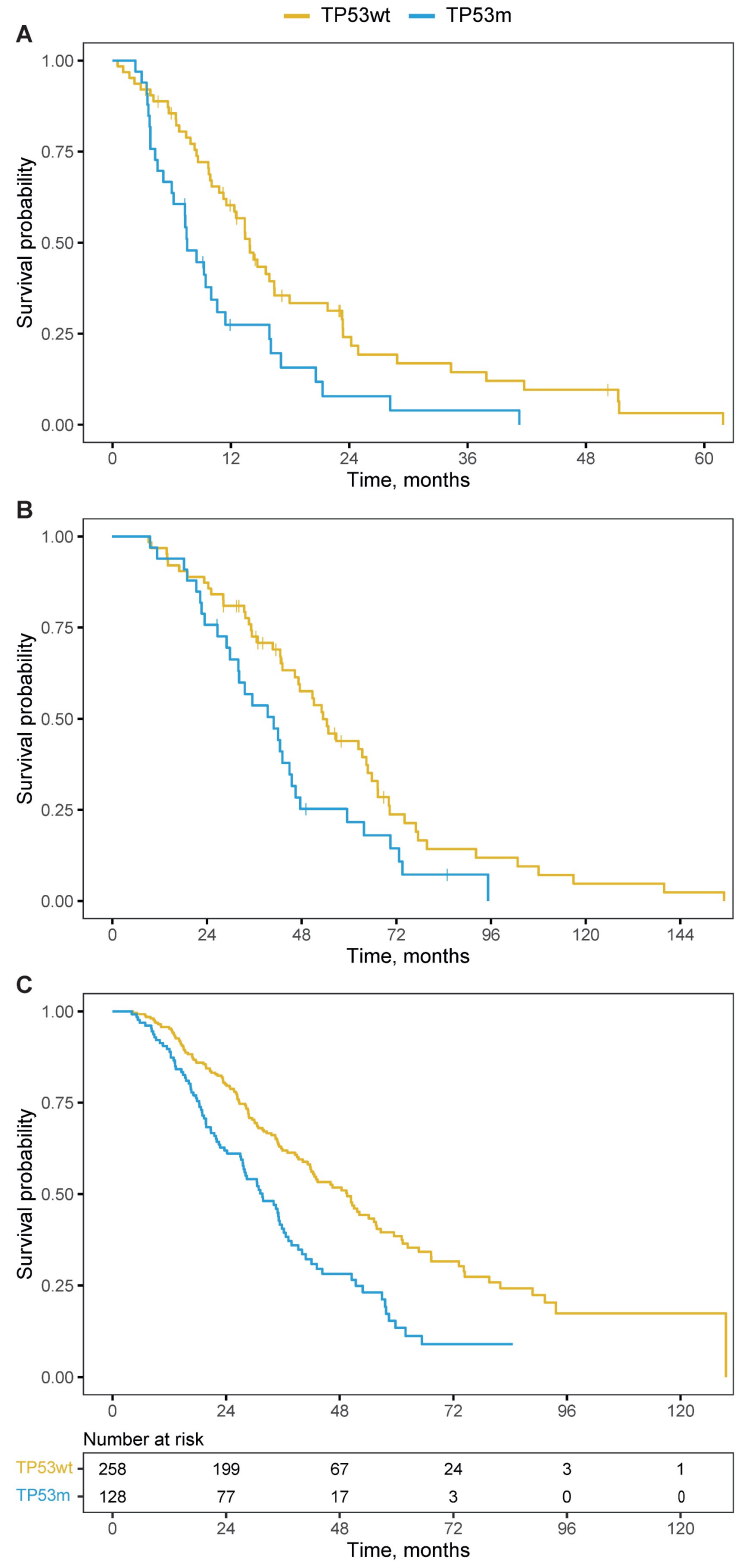
Kaplan-Meier curves for overall survival per *TP53*-mutational status. **A.** from initiation of PSMA-RLT. **B.** from castration-resistance. **C.** from castration-resistance for a non-PSMA-RLT-treated cohort, including a table presenting the number of patients at risk. Abbreviations: TP53wt, *TP53* wildtype; TP53m, *TP53* mutated.

The TP53m subgroup had a significantly shorter OS when compared to the TP53wt subgroup. From initiation of PSMA-RLT, the median OS was 7.6 versus 13.9 months (HR 2.0; 95%CI 1.3 - 3.2; *P*=0.003; Figure 3A) and remained significant when corrected for the line of treatment in which PSMA-RLT was initiated (HR 2.1; 95%CI 1.3 - 3.3; *P*=0.003). From moment of castration-resistance, the OS was 40.9 months for the TP53m subgroup and 53.4 months for the TP53wt subgroup (HR 1.7; 95%CI 1.1 - 2.8; *P*=0.019; Figure 3B).

#### Beyond PSMA-RLT

The prognostic power of *TP53* loss-of-function alterations is well known and observed for several therapies for mCRPC. To validate the importance of *TP53* mutational status beyond PSMA-RLT, we constructed a cohort of 386 mCRPC patients sequenced with the same inhouse targeted sequencing panel or whole-genome sequencing as the main study population but did not receive PSMA-RLT. The median OS from moment of castration-resistance was 41.2 months (95%CI 35.9 - 48.4). The TP53m patients (n=128) had a median OS of 31.7 months compared to 49.5 months for the 258 TP53wt patients (HR 1.9; 95%CI 1.5 - 2.5; *P*<0.001; Figure 3C). This is in line with the OS difference in the patients treated with PSMA-RLT.

### Enrichment analysis in CRPC patients treated with PSMA-RLT

For 21 out of 96 patients from the main analysis, RNA sequencing was performed on tissues obtained before initiation PSMA-RLT. One patient (study ID 28) received ^177^Lu-PSMA followed by ^225^Ac-PSMA with tissue obtained in between. For this specific analysis, we ensured that all RNA sequencing was performed on pre-treatment tissue, and therefore for study ID 28 we only assessed response to the second PSMA-RLT (^225^Ac-PSMA).

#### Enrichment analysis

**Figure 4:**
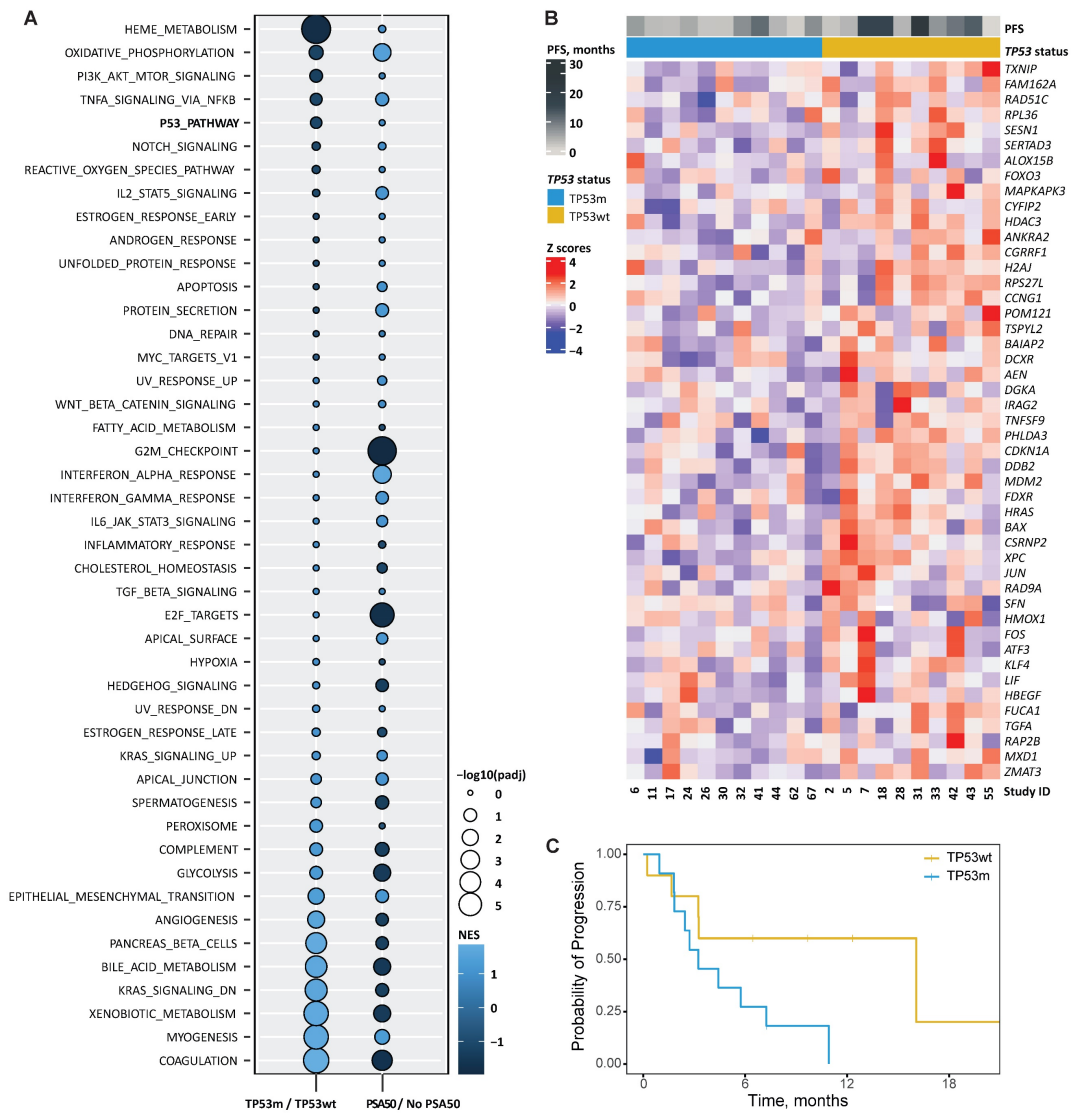
** A.** Bubble plot showing hallmarks of cancer signatures enriched (light blue) or decreased (dark blue) when comparing gene expression of patients with and without *TP53* loss-of-function alterations (TP53m/TP53wt, respectively) and with and without ≥50% PSA decline (PSA50). The colour in the graph represents the normalised enrichment score (NES) and the size the false discovery rate-adjusted *P*-value (padj). **B.** Heatmap showing the relative change in mRNA expression of genes from the signature HALLMARK P53 PATHWAY that were enriched in the comparison TP53m/TP53wt (n=47) across patients. Rows show Z scores of normalised, log2-transformed values. Progression-free survival (PFS) and *TP53* status for each patient is depicted.** C.** Kaplan-Meier curves per *TP53*-mutational status for the progression-free survival on PSMA-RLT for the 21 patients who underwent RNA sequencing.

For the GSEA, we used the dichotomic endpoint PSA50 instead of PFS, as this generally correlates with PFS in mCRPC 34, 35. Figure 4A visualises the GSEA based on TP53m over TP53wt and PSA50 over no PSA50. Several signatures were found commonly enriched in patients with PSA50 and TP53wt status. Among these, we found signatures involved in transcription factor activity, like NF-kB signalling in response to TNFα, the PI3K/AKT/MTOR pathway, NOTCH signalling, the p53 pathway, IL2/STAT5 signalling, an androgen responsive and an early oestrogen responsive gene signature, and MYC signalling. In contrast, besides E2F signalling and genes downregulated by KRAS activation, signatures commonly enriched in TP53m patients without PSA50 were constituted mainly by genes involved in biological processes like the development of skeletal muscle, genes encoding components of the blood coagulation system, genes associated with metabolism of xenobiotics, and bile acids and salts, genes encoding components of the complement immune system, and genes regulating glycolysis (Figure 4A). The expression of the 47 genes driving the enrichment in the TP53m/TP53wt comparison is visualised in Figure 4B. Their expression separated patients based on PFS, in line with the main analysis. TP53m patients in this subset had a significantly shorter PFS on PSMA-RLT (*P*=0.045; Figure 4C).

#### p53 pathway genes association with PFS

**Figure 5:**
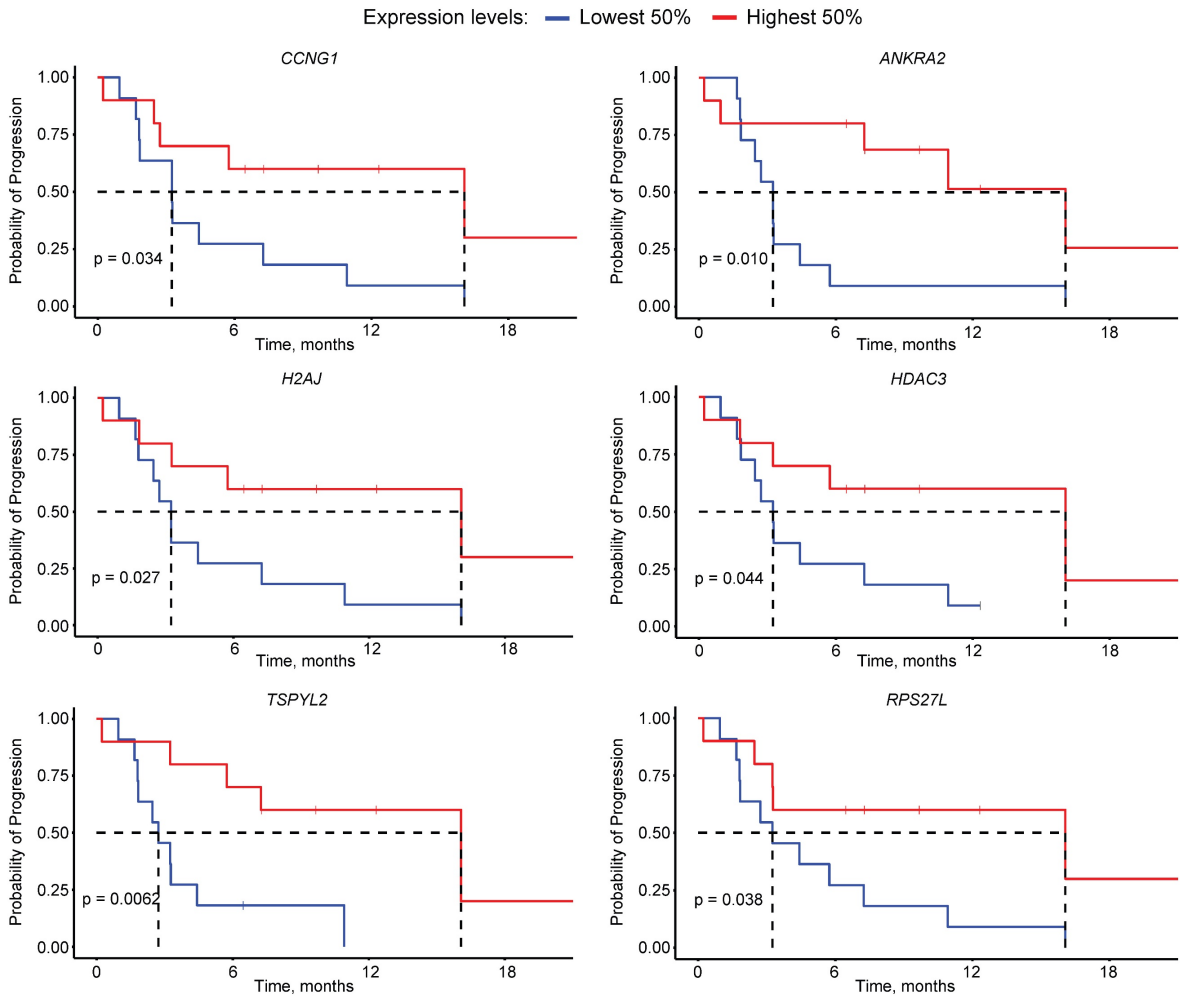
Kaplan-Meier curves for the progression-free survival based on the expression of *CCNG1, ANKRA2, H2AJ, HDAC3, TSPYL2* and* RPS27L*.

To identify possible drivers of poor outcome to PSMA-RLT among the target genes of p53, we first selected the 18 genes commonly down-regulated in TP53m patients without PSA50 (Supplementary Table 5). Survival analysis determined that the expression of six of these genes, namely *CCNG1*, *ANKRA2*, *H2AJ*, *HDAC3*, *TSPYL2* and *RPS27L*, significantly affected the PFS (*P*<0.05), where the low expression of each gene was independently associated with a short PFS (Figure 5). From these, *CCNG1*, *ANKRA2* and *RPS27L* are known p53 targets 36, whereas *TSPYL2* is vital for effective p53 activation 37. The p53 target genes *FUCA1*, *RAP2B* and *SESN1* showed a similar trend but did not reach statistical significance (Supplementary Figure 3) 37.

## Discussion

In this pre-defined retrospective cohort study, we showed that mCRPC patients with *TP53* loss-of-function alterations generally respond worse to PSMA-RLT in terms of PFS, biochemical response, and OS when compared to patients without *TP53* loss-of-function alterations. In multivariable analyses with other canonical oncogenic pathways and HRRm, *TP53* status was independently associated with response. In multivariable analyses with known prognostic factors, LDH was significantly associated with response alongside *TP53* status. To our knowledge, this is the largest published molecularly profiled mCRPC population treated with PSMA-RLT.

Previous, mostly small, retrospective studies have failed to establish consensus regarding *TP53* mutational status as predictor for response to PSMA-RLT. Vanwelkenhuyzen *et al*. included 46 mCRPC patients who received ^177^Lu-PSMA and analysed blood for qualitative circulating tumour DNA analysis. In the 39 patients with detectable circulating tumour DNA, *TP53* mutational status was not associated with ^177^Lu-PSMA outcomes. Notably, the seven patients without detectable circulating tumour DNA were classified as lacking (*TP53*) genetic alterations. Another study, which included only 15 molecularly profiled mCRPC patients, identified two patients with a *TP53* alteration, both did not respond to PSMA-RLT 20. Kratochwil *et al.* described that six out of seven poor responders were associated with enhanced p53 signalling: 3/7 harboured a *TP53* alteration, 2/7 a *ATM* alteration and 2/7 a *CHEK2* alteration (one with a concurrent *TP53* alteration) 21.

It was considered that the observed difference in PFS per *TP53* status might not solely be attributed to *TP53*, but instead could be attributed to the presence of AVPC, characterised by compound genomic alterations in *RB1*, *TP53*, and/or *PTEN* 38. AVPC, which exhibits features of small cell (neuroendocrine) prostate cancer, can lead to PSMA suppression, potentially reducing the effectiveness of PSMA-RLT 39-41. However, our data suggests that *TP53* status is a stronger predictor of PSMA-RLT outcomes than the molecular signature of AVPC.

The impact of TP53m on response to PSMA-RLT was compared to other genetic subgroups or prognostic variables. Apart from TP53m, none of the other genetic subgroups was associated with response to PSMA-RLT. In contrast to our findings, the aforementioned Vanwelkenhuyzen *et al*. identified pathogenic alterations in the PI3K pathway as most strongly associated with a shorter PFS 19. De Giorgi *et al*., found *AR* amplifications to be linked with a shorter PFS 19, 42. In our cohort, among 29 patients with *AR* amplifications, the median PFS was 5.4 months compared to 7.1 months for those without *AR* amplifications but did not reach statistical significance (*P*=0.51, data not presented). Raychaudhuri *et al*. reported a significantly higher PSA50 rate for patients with HRRm 43. However, in our cohort, HRRm did not appear to have any discernible impact on the response to PSMA-RLT. Handke et al. conducted a transcriptome analysis on 23 patients, revealing an association between PD-L2 expression and response to PSMA-RLT. In our subgroup of 21 patients with available transcriptome data, however, PD-L2 expression did not correlate with PFS (P=0.64, data not presented). The only known prognostic variable, alongside *TP53* status, significantly associated with response on PSMA-RLT was LDH. As described in two large meta-analyses, high LDH levels are associated with shorter OS and PFS across therapies for mCRPC 44, 45. For PSMA-RLT specifically, LDH is more strongly associated with progression on ^177^Lu-PSMA than ALP or PSA 46, 47.

As p53 is a transcription factor, its functionality can be measured by the expression of its target genes. Within a subset of 21 patients, we identified gene expression signatures associated with both *TP53* mutational status and biochemical response. While the KRAS pathway was enriched in TP53m patients without PSA50, a TNFα signature was enriched in TP53wt patients with PSA50*.* Although the *KRAS* gene is not commonly aberrant in metastatic prostate cancer (7%), deregulation of RAS proteins signalling has been reported and has tumour-promoting activity 48. Depending on the biological context, TNFα can have two distinct roles in prostate cancer. In androgen-dependent tumours, TNFα signalling can drive the progression to castration-resistance 49. On the other hand, and in line with our findings, in mCRPC TNFα has demonstrated to have an anti-tumour activity, by being effective in destroying tumour vasculature and stimulating anti-tumour immunity. Moreover, TNFα sensitises prostate cancer cells to ionizing radiation 50.

The enrichment analysis also identified 18 genes constituting the MSigDB p53 signature from the GSEA, whose transcript expression was markedly lower in TP53m patients without a PSA50. The low expression of six of these genes, namely* CCNG1, ANKRA2, H2AJ, HDAC3, TSPYL2* and* RPS27L,* resulted in a significantly poorer PFS in our cohort of mCRPC patients. High expression of *TSPYL2* and *RPS27L* correlate with better cancer prognosis across various cancer types 37, 51. These genes are involved in inducing senescence, which in prostate cancer, upon ionizing radiation, is mainly mediated through p53 37, 52, 53. Additionally, *TSPYL2* regulates p53 acetylation and p53-dependent cell death, potentially contributing to its tumour-suppressing activity 37, 54. CCNG1 and ANKRA2 expression changes in response to ionizing radiation exposure, potentially serving as biomarkers 55. In contrast to our results, lower expression of *HDAC3* led to increased sensitivity to ionizing radiation in preclinical models 56.

Few patients, presumed to have a loss-of-function alteration in *TP53*, did show relatively high target gene expression. Downregulation of p53-mediated signalling requires inadequate p53 tetramerization, through homozygous loss or pathogenic mutations, even without loss of heterozygosity due to the dominant negative effect of most *TP53* mutations 57, 58. These discrepancies may be due to functional tetramerization by amplification of the wildtype allele or mutational exceptions.

Our analyses consistently identify TP53m as prognostic for poor response to PSMA-RLT. However, it may also have predictive value. The comparable OS deficit of TP53m patients in the populations treated with and without PSMA-RLT suggests that *TP53* status is prognostic rather than predictive for response on PSMA-RLT. Yet, the rationale for *TP53* alterations as a predictive factor cannot be overseen. Evidence from preclinical studies indicates p53 upregulation in response to PSMA-RLT and reduced sensitivity in *TP53*^-/-^ tumours 17. Additionally, *TP53* loss-of-function alterations are often suggested as drivers of resistance to ionizing radiation, suggesting a predictive role 59-61. The predictive value of *TP53* mutations may extend to other therapies for mCRPC, with conflicting findings regarding response to taxanes or ARSIs based on *TP53* status 62-65. Preliminary data from the first prospective trial evaluating standard of care treatment based on *TP53* status have not shown differences in responses to ARSIs or taxanes 66.

This study has several limitations that should be acknowledged. Firstly, due to its retrospective nature, there is missing data, leading to possible bias and reducing the power of the multivariable models. While this is the largest published population of its kind, the relatively low patient number means that this study may be underpowered to find associations with less prevalent molecular subgroups. Additionally, the cohort is heterogeneous as patients received different radionuclides and different PSMA ligands (PSMA-I&T or PSMA-617). The lack of standardised guidelines for PSMA-RLT administration throughout most of the inclusion period and delivery problems led to varying dosages per cycle and different number of administered cycles, which may have influenced treatment outcomes. In some cases, disease progression may have occurred due to postponed cycles, and patients experienced repeated responses after receiving subsequent cycles. Further limitations include imbalanced characteristics between TP53wt and TP53m patients, such as concurrent ARSI, and variations in biopsy timing relative to PSMA-RLT initiation. Although TP53 alterations are well-established as early and truncal events 67, 68, patients who underwent molecular profiling solely on archived primary tissue from localised prostate cancer are at small risk of underrepresentation of TP53 alterations due to intratumoural heterogeneity 69. Also, the SUVmean of all lesions probably offers a more accurate assessment than the SUVmax of the hottest lesion for measuring PSMA expression 12, 70-74.

## Conclusion

This study, describing the largest cohort of PSMA-RLT treated and molecularly profiled patients with mCRPC, confirms the preclinical indication that *TP53* loss-of-function alterations are indicators for an unfavourable response on PSMA-RLT. No other canonical oncogenic or tumour suppressive pathway was associated with PSMA-RLT response. These results underscore the potential of molecular tumour profiling of mCRPC patients to personalise treatment plans with the goal of limiting unnecessary toxicities and improving OS and quality of life.

## Supplementary Material

Supplementary figures and tables.

## Figures and Tables

**Table 1:** Diagnostic, baseline, and treatment variables of the study population

Variable	N	Missing	All	TP53wt	TP53m	P-value
			Number of patients (valid %) or Median [interquartile range]			
**Diagnostic variables**						
ISUP-GGS	95	1				0.733
1			9 (9.5)	7 (11.3)	2 (6.1)	
2			13 (13.7)	8 (12.9)	5 (15.2)	
3			10 (10.5)	6 (9.7)	4 (12.1)	
4			23 (24.2)	17 (27.4)	6 (18.2)	
5			40 (42.1)	24 (38.7)	16 (48.5)	
Metastatic at diagnosis	95	1				0.832
No			50 (52.6)	32 (51.6)	18 (54.6)	
Yes			45 (47.4)	30 (48.4)	15 (46.5)	
Initial PSA level (µg/L)	83	13	41.00 [10.8 - 131.0]	48.00 [21.4 - 142.0]	16.75 [8.5 - 86.5]	0.053
Age at initial diagnosis, years	96	0	61.8 [56.2 - 67.0]	61.6 [55.3 - 67.0]	63.3 [58.5 - 66.2]	0.287
Age at CRPC, years	96	0	66.3 [60.4 - 72.2]	66.1 [60.3 - 72.1]	66.3 [62.7 - 73.1]	0.459
Time to CRPC, months	96	0	15.5 [10.0 - 30.0]	16.0 [10.5 - 29.0]	14.0 [9.7 - 31.7]	0.948
**At start of PSMA-RLT**						
Line of therapy for CRPC	96	0	4 3 - 5	4 3 - 5	4 3 - 5	0.903
PSMA-PET characteristics						
SUVmax	80	16	55.1 [29.8 - 73.6]	57.6 [30.6 - 71.1]	54.1 [29.6 - 84.8]	0.904
Bone metastases	93	3	85 (91.4)	54 (90.0)	31 (93.9)	0.707
bone only			20 (21.5)	15 (25.0)	5 (15.2)	
Visceral metastases	93	3	27 (29.0)	18 (30.0)	9 (27.3)	0.816
Laboratory variables						
PSA (µg/L)	94	2	233.0 [75.6 - 551.5]	270.00 [79.6 - 794.7]	163.76 [59.3 - 528.5]	0.280
ALP (U/L)	89	7	138.0 [90.0 - 291.0]	140.5 [91.8 - 310.3]	136.0 [91.5 - 266.0]	0.711
LDH (U/L)	87	9	258.0 [211.0 - 355.2]	249.5 [207.0 - 349.3]	272.0 [219.5 - 394.5]	0.338
HB (mmol/L)	85	11	7.4 [6.5 - 8.2]	7.3 [6.5 - 8.2]	7.7 [6.5 - 8.2]	0.887
**PSMA-RLT**						
Type of radioligand	96	0				0.279
^177^Lutetium			76 (79.2)	52 (82.5)	24 (72.7)	
^225^Actinium			7 (7.3)	5 (7.9)	2 (6.1)	
Tandem			13 (13.5)	6 (9.5)	7 (21.2)	
Cycles of PSMA-RLT	96	0	3 2 - 5	4 2 - 6	3 2 - 3	**0.028**
≥4 cycles			42 (43.8)	34 (54.0)	8 (24.2)	
Concurrent therapy	96	0				**0.006**
Enzalutamide			10 (10.42)	2 (3.17)	8 (24.24)	
Abiraterone			7 (7.29)	5 (7.94)	2 (6.06)	
None			79 (82.29)	56 (88.89)	23 (69.70)	

*P*-values in bold are considered significant. Abbreviations: ALP, alkaline phosphatase; CRPC, castration-resistant prostate cancer; HB, haemoglobin; ISUP-GGS, International Society of Urological Pathology Gleason grading system; LDH, lactate dehydrogenase; PSA, prostate specific antigen; PSMA-RLT, prostate-specific membrane antigen-targeting radioligand therapy; SUVmax, maximal standardised uptake value; TP53m, *TP53* mutated; TP53wt, *TP53* wildtype.

**Table 2:** Univariable and multivariable analysis of potential prognostic molecular subgroups for progression-free survival on PSMA-RLT.

Molecular subgroup	Effect of alteration	Univariable analysis			Multivariable analysis	
		HR [95%CI]	*P*-value		HR [95%CI]	*P*-value
*TP53*	Inactivation	2.21 [1.40-3.49]	**<0.001**		2.53 [1.52-4.22]	**<0.001**
*AR*	Hyperactivation	0.86 [0.70-1.06]	0.155		0.85 [0.69-1.05]	0.130
*MYC*	Hyperactivation	1.18 [0.57-2.45]	0.661		0.61 [0.26-1.45]	0.263
PI3K	Hyperactivation	1.13 [0.72-1.78]	0.597		1.13 [0.70-1.82]	0.628
*BRCA1*/*2*	Inactivation	1.04 [0.57-1.88]	0.905		0.90 [0.43-1.89]	0.776
HRR	Inactivation	0.96 [0.62-1.49]	0.847		1.07 [0.61-1.88]	0.811
MAPK	Hyperactivation	1.05 [0.38-2.89]	0.921		0.99 [0.33-3.02]	0.987

*P*-values in bold are considered significant. Abbreviations: CI, confidence interval; HR, hazard ratio; HRR, homologous recombination repair.

## Abbreviations

^177^Lu: lutetium-177
^225^Ac: actinium-225
ARSI: androgen receptor signalling inhibitor
AVPC: aggressive variant prostate cancer
bPFS: biochemical PFS
BRCAm: *BRCA1* or *BRCA2* mutated
CI: confidence interval
GSEA: gene-set enrichment analysis
HR: hazard ratio
HRRm: impairment of homologous recombination repair
LDH: lactate dehydrogenase
mCRPC: metastatic castration-resistant prostate cancer
OS: overall survival
PFS: progression-free survival
PSA50: ≥50% PSA decline
PSMA: prostate specific membrane antigen
RLT: radioligand therapy
SUVmax: maximum standardised uptake values
TP53m: *TP53* mutated
TP53wt: *TP53* wild-type
WGS: whole-genome sequencing
