# Supplementary material for: Impact of TP53 loss-of-function alterations on the response to PSMA radioligand therapy in metastatic castration-resistant prostate cancer patients
Source: Theranostics. 2024 Aug 1;14(12):4555–69. doi: 10.7150/thno.96322 (PMC11373632; doi:10.7150/thno.96322)
Supplement: Supplementary file 1 — Supplementary figures and tables. [file thnov14p4555s1.pdf]

Supplementary data to:

Impact of *TP53* Loss-of-Function Alterations on the Response to PSMA Radioligand Therapy in Metastatic Castration-Resistant Prostate Cancer Patients

Peter H.J. Slootbeek [1], María Victoria Luna-Velez [2], Bastiaan M. Privé [3], Maarten J. van der Doelen [2], Iris S.H. Kloots [1], Samhita Pamidimarri Naga [1], Hilde E. Onstenk [1], James Nagarajah [3, 4], Harm Westdorp [1], Inge M. van Oort [2], Leonie I. Kroeze [5], Jack. A. Schalken [2], Haiko J. Bloemendal [1], Niven Mehra [1, *]

**Supplementary Table 1.**	Individual data on activity and interval per cycle of PSMA-RLT.
**Supplementary Table 2.**	Multivariable analysis of *TP53*-mutational status and known prognostic factors for progression-free survival on PSMA-RLT.
**Supplementary Table 3.**	Biochemical response per mutational status of canonical oncogenic or tumour-suppressive pathways.
**Supplementary Table 4.**	Multivariable analysis for change of biochemical progression per mutational status of canonical oncogenic or tumour-suppressive pathways.
**Supplementary Table 5.**	Genes from the signature HALLMARK P53 PATHWAY driving enrichment in PSA50/No PSA50 and decreased in TP53m/TP53wt.
**Supplementary Figure 1.**	Kaplan-Meier curves for the progression-free survival per AVPC and impaired p53 signalling.
**Supplementary Figure 2.**	Kaplan-Meijer curves for the biochemical progression-free survival probability per *TP53*-mutational status.
S**upplementary Figure 3.**	Kaplan-Meier curves for the progression-free survival based on the expression of *FUCA1, RAP2B,* and *SESN1*.

Supplementary Table 1. Individual data on activity and interval per cycle of PSMA-RLT.

Study ID	*TP53* status	Number of cycles	Mean weeks in between cycles	Total ^{177}Lu activity (GBq)	Mean ^{177}Lu acitvity per cycle (GBq)	Total ^{225}Ac activity (MBq)	Mean ^{225}Ac acitvity per cycle (MBq)
1	TP53wt	3	5,0			24,0	8,0
2	TP53wt	1				8,0	8,0
3	TP53wt	4	8,4	15,0	3,8	17,5	4,4
4	TP53m	2	5,5	13,9	7,0	2,2	1,1
5	TP53wt	2	4,6	14,9	7,5		
6	TP53m	2	3,9			14,0	7,0
7	TP53wt	6	10,4	24,0	4,0	26,0	4,3
8	TP53m	3	3,4	21,1	7,0	4,1	1,4
9	TP53wt	6	5,7	44,4	7,4		
10	TP53m	2	2,4	16,7	8,4		
11	TP53m	2	3,6	14,7	7,4		
12	TP53m	6	7,3	36,2	6,0		
13	TP53wt	3	5,7	18,5	6,2		
14	TP53m	3	5,7	14,0	4,7	8,0	2,7
15	TP53wt	3	5,2			20,1	6,7
16	TP53wt	5	5,9	30,2	6,0		
17	TP53m	2	3,5	11,4	5,7		
18	TP53wt	7	31,0	13,4	1,9	35,0	5,0
19	TP53wt	4	8,1			31,4	7,9
20	TP53wt	2	4,0	12,0	6,0		
21	TP53wt	2	3,4	10,7	5,4		
22	TP53wt	2	4,6	5,0	2,5	12,4	6,2
23	TP53m	3	3,8	19,8	6,6		
24	TP53m	4	4,8	29,6	7,4	2,2	0,6
25	TP53wt	4	5,6	24,0	6,0		
26	TP53m	2	4,0	16,5	8,3	2,0	1,0
27	TP53m	3	7,0			18,8	6,3
28	TP53wt	2	3,9	10,0	5,0		
29	TP53wt	4	4,8	24,4	6,1		
30	TP53m	2	3,5	12,0	6,0		
31	TP53wt	3	6,4			20,0	6,7
32	TP53m	3	13,3	12,2	4,1		
33	TP53wt	4	5,0	24,1	6,0		
34	TP53wt	4	5,6	24,5	6,1		
35	TP53wt	6	6,9	35,8	6,0		
36	TP53wt	4	4,0	28,2	7,1		
37	TP53wt	3	7,0	18,3	6,1		
38	TP53wt	4	5,3	24,0	6,0		
39	TP53m	2	4,9	15,4	7,7		
40	TP53wt	2	4,5	14,0	7,0	14,5	7,3
41	TP53m	6	5,0	44,4	7,4		
42	TP53wt	6	5,0	44,4	7,4		
43	TP53wt	6	5,0	44,4	7,4		
44	TP53m	3	4,0	22,2	7,4		
45	TP53wt	6	5,6	44,4	7,4		
46	TP53wt	6	5,2	44,4	7,4		
47	TP53m	6	5,0	44,4	7,4		
48	TP53wt	6	5,1	44,4	7,4		
49	TP53wt	3	4,0	22,2	7,4		
50	TP53wt	6	5,0	44,4	7,4		
52	TP53wt	3	4,1	22,2	7,4		
53	TP53m	4	4,4	29,6	7,4		
54	TP53wt	2	3,0	14,8	7,4		
55	TP53wt	1		7,4	7,4		
56	TP53wt	3	20,6	22,2	7,4		
57	TP53wt	6	4,9	44,4	7,4		
59	TP53m	6	5,7	44,4	7,4		

60	TP53m	2	3,0	14,8	7,4		
61	TP53wt	3	5,7	25,5	8,5		
62	TP53m	1		6,0	6,0		
63	TP53wt	1		6,7	6,7		
64	TP53wt	3	4,7	18,0	6,0		
65	TP53m	2	3,0	13,7	6,9		
66	TP53wt	2	7,3	11,4	5,7	4,0	2,0
67	TP53m	2	3,9	13,6	6,8		
68	TP53wt	2	3,0	14,8	7,4		
69	TP53m	6	5,1	44,4	7,4		
101	TP53wt	5	10,1				
102	TP53m	2	3,0	14,8	7,4		
103	TP53wt	2	3,4	14,9	7,5		
104	TP53m	3	4,1	22,2	7,4		
105	TP53wt	1		7,4	7,4		
106	TP53wt	6	6,0	44,4	7,4		
107	TP53wt	3	4,0	22,2	7,4		
108	TP53wt	6	5,7	44,4	7,4		
109	TP53m	2	1,0				
110	TP53wt	6	6,0	44,4	7,4		
111	TP53wt	4	4,5				
112	TP53wt	1		7,4	7,4		
113	TP53wt	2	3,0	14,8	7,4		
114	TP53wt	6	6,2	44,4	7,4		
115	TP53m	3	6,0	14,0	4,7	10,0	3,3
116	TP53wt	6	6,2	44,4	7,4		
117	TP53wt	5	5,8	36,6	7,3		
118	TP53m	5	7,0	38,4	7,7	22,8	4,6
119	TP53m	3	6,3	20,4	6,8		
120	TP53wt	4	6,5	27,2	6,8		
121	TP53wt	5	4,4	37,0	7,4		
123	TP53wt	4	6,2	27,2	6,8		
124	TP53wt	6	5,5	44,4	7,4		
125	TP53wt	2	4,0	17,8	8,9		
126	TP53m	2	3,9	13,6	6,8		
127	TP53m	1					
128	TP53wt	2	1,0	14,8	7,4		
129	TP53wt	4	4,5	29,6	7,4		
130	TP53wt	5	1,2	37,0	7,4		

^{177}Lu, ^{177}Lutetium; ^{225}Ac, ^{225}Actinium; GBq, gigaBecquerel; MBq, megaBecquerel; TP53m, *TP53* mutated; TP53wt, *TP53* wildtype.

Supplementary Table 2. Multivariable analysis of *TP53*-mutational status and known prognostic factors for progression-free survival on PSMA-RLT.

Variables	HR [95%CI]	*P*-value
TP53m	2.27 [1.27-4.05]	**0.005**
Line of therapy	1.09 [0.93-1.28]	0.298
Time from initial diagnosis to castration-resistance	1.00 [0.99-1.01]	0.999
SUVmax of highest lesions on baseline PSMA-PET scan	1.00 [0.99-1.01]	0.703
Presence of visceral metastasis on baseline PSMA-PET scan	0.85 [0.56-1.58]	0.607
Type of radioligand therapy	0.76 [0.52-1.11]	0.151
Laboratory variables at baseline:		
PSA	1.00 [1.00-1.00]	0.133
LDH	1.00 [1.00-1.01]	**0.001**
HB	0.92 [0.76-1.12]	0.414
ALP	1.00 [1.00-1.00]	0.163

ALP, alkaline phosphatase; CI, confidence interval; HB, haemoglobin; HR, hazard ratio; LDH, lactate dehydrogenase; PSA, prostate specific antigen; SUVmax, maximum standardised uptake values.

Supplementary Table 3. Biochemical response per mutational status of canonical oncogenic or tumour-suppressive pathways.

Genetic subgroup	Effect of alteration	Median PSA response		Patients with PSA50	
		Mutated vs. WT	*P*-value	Mutated vs. WT	*P*-value
TP53	Inactivation	-55% vs. -75%	**0.012**	45% vs. 56%	0.569
AR	Hyperactivation	-70% vs. -61%	0.294	69% vs. 57%	0.380
MYC	Hyperactivation	-66% vs. -65%	0.306	57% vs, 61%	1.000
PI3K	Hyperactivation	-64% vs. -68%	0.456	62% vs. 61%	0.980
BRCA1/2	Inactivation	-35% vs. -66%	0.971	45% vs. 63%	0.327
HRR	Inactivation	-69% vs. -64%	0.804	59% vs. 62%	0.825
MAPK	Hyperactivation	-85% vs. -64%	0.214	100% vs. 51%	0.154

HRR, homologous recombination repair; PSA, ≥50% PSA decline; PSA, prostate specific antigen; WT, wildtype.

Supplementary Table 4. Multivariable analysis for change of biochemical progression per mutational status of canonical oncogenic or tumour-suppressive pathways.

Genetic subgroup	Univariable analysis	
	HR [95%CI]	*P*-value
TP53	2.53 [1.52-4.22]	**<0.001**
AR	0.85 [0.69-1.05]	0.130
MYC	0.61 [0.26-1.45]	0.263
PI3K	1.13 [0.70-1.82]	0.628
BRCA1/2	0.90 [0.43-1.89]	0.776
HRR	1.07 [0.61-1.88]	0.811
MAPK	0.99 [0.33-3.02]	0.987

CI, confidence interval; HR, hazard ratio; HRR, homologous recombination repair.

Supplementary Table 5. Genes from the signature HALLMARK P53 PATHWAY driving enrichment in PSA50/No PSA50 and decreased in TP53m/TP53wt.

**TP53m/TP53wt**		**PSA50/No PSA50**		**Intersect**
ALOX15B	*RAD9A*	*TNNI1*	*LIF*	FOS
FOS	*RAD51C*	*FOS*	*IRAG2*	SESN1
SESN1	*H2AJ*	*CTSF*	*HDAC3*	TXNIP
PHLDA3	*CSRNP2*	*TP63*	*TGFA*	RPS27L
TXNIP	*SERTAD3*	*INHBB*	*RPL18*	TGFA
FAM162A	*RPL36*	*SESN1*	*RB1*	TSPYL2
KLF4	*HDAC3*	*HBEGF*	*TSC22D1*	MAPKAPK3
JUN	*CYFIP2*	*H2AJ*	*TSPYL2*	LIF
RPS27L	*BAX*	*BTG2*	*VDR*	ATF3
TGFA	*HRAS*	*LDHB*	*RAP2B*	IRAG2
TSPYL2	*POM121*	*CYFIP2*	*ACVR1B*	CCNG1
DDB2	*FOXO3*	*RACK1*	*VWA5A*	HBEGF
MAPKAPK3	*FDXR*	*TAP1*	*COQ8A*	FUCA1
CDKN1A	*ZMAT3*	*GM2A*	*ABCC5*	H2AJ
LIF	*RAP2B*	*RRAD*	*ITGB4*	HDAC3
DCXR	*TNFSF9*	*MAPKAPK3*	*EPHX1*	CYFIP2
MDM2	*ANKRA2*	*STEAP3*	*ATF3*	RAP2B
ATF3	*CGRRF1*	*TGFB1*	*NUDT15*	ANKRA2
BAIAP2	*MXD1*	*CCNG1*	*TXNIP*	
IRAG2	*XPC*	*FUCA1*	*ANKRA2*	
DGKA		*S100A4*	*PTPRE*	
CCNG1		*SOCS1*	*SPHK1*	
HBEGF		*RPS27L*	*KRT17*	
AEN		*EPHA2*	*RPS12*	
FUCA1		*EI24*	*HEXIM1*	
HMOX1		*SAT1*	*PVT1*	
SFN		*BMP2*		

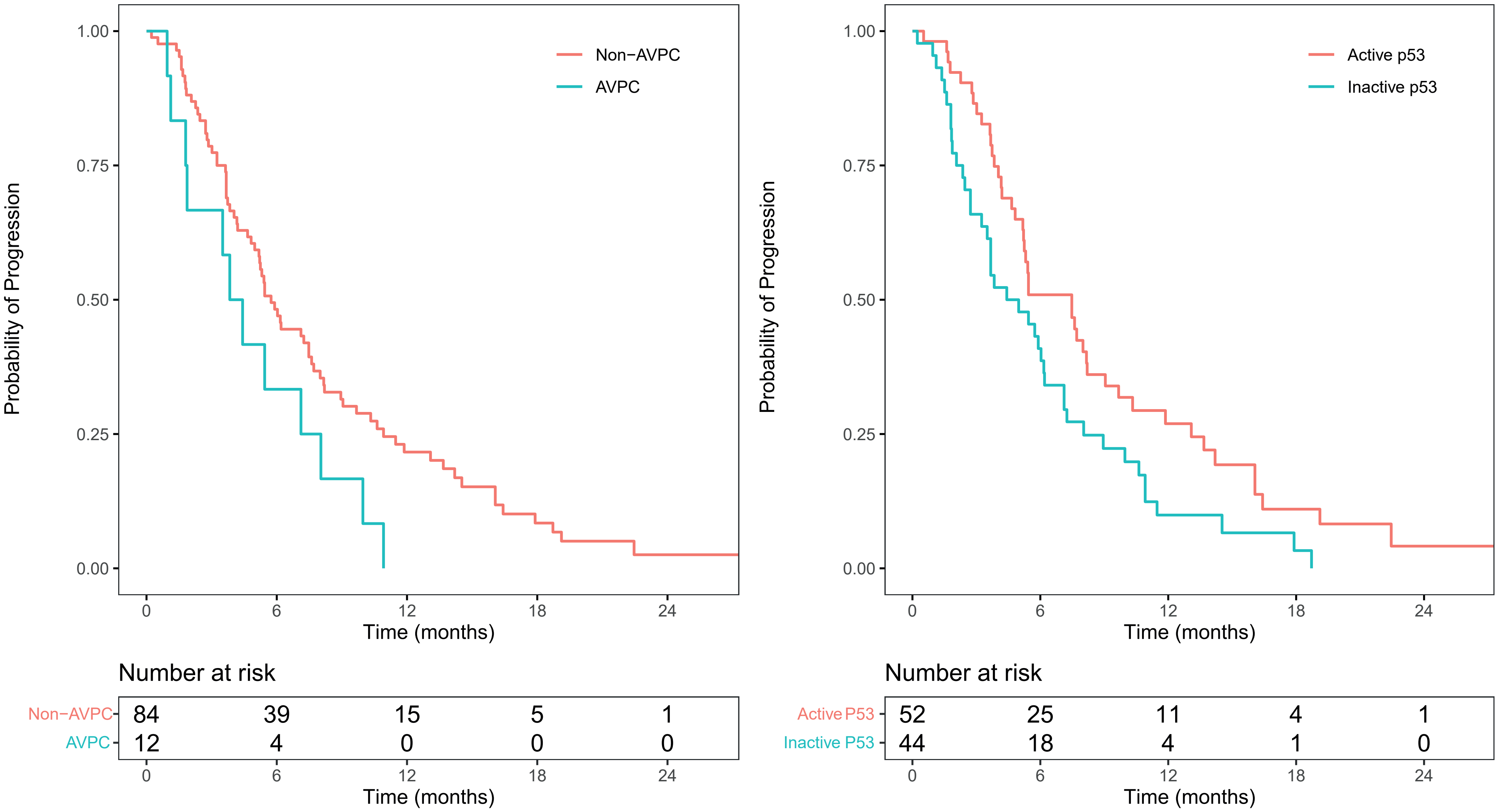

Kaplan-Meier curves for the progression-free survival, including a table presenting the number of patients at risk for: (**Left**) patients with and without an aggressive variant prostate cancer (AVPC) molecular signature and (**Right**) patients with and without an impaired p53 signalling based on loss-of-function alterations in TP53, ATM, CHEK1, or CHEK2.

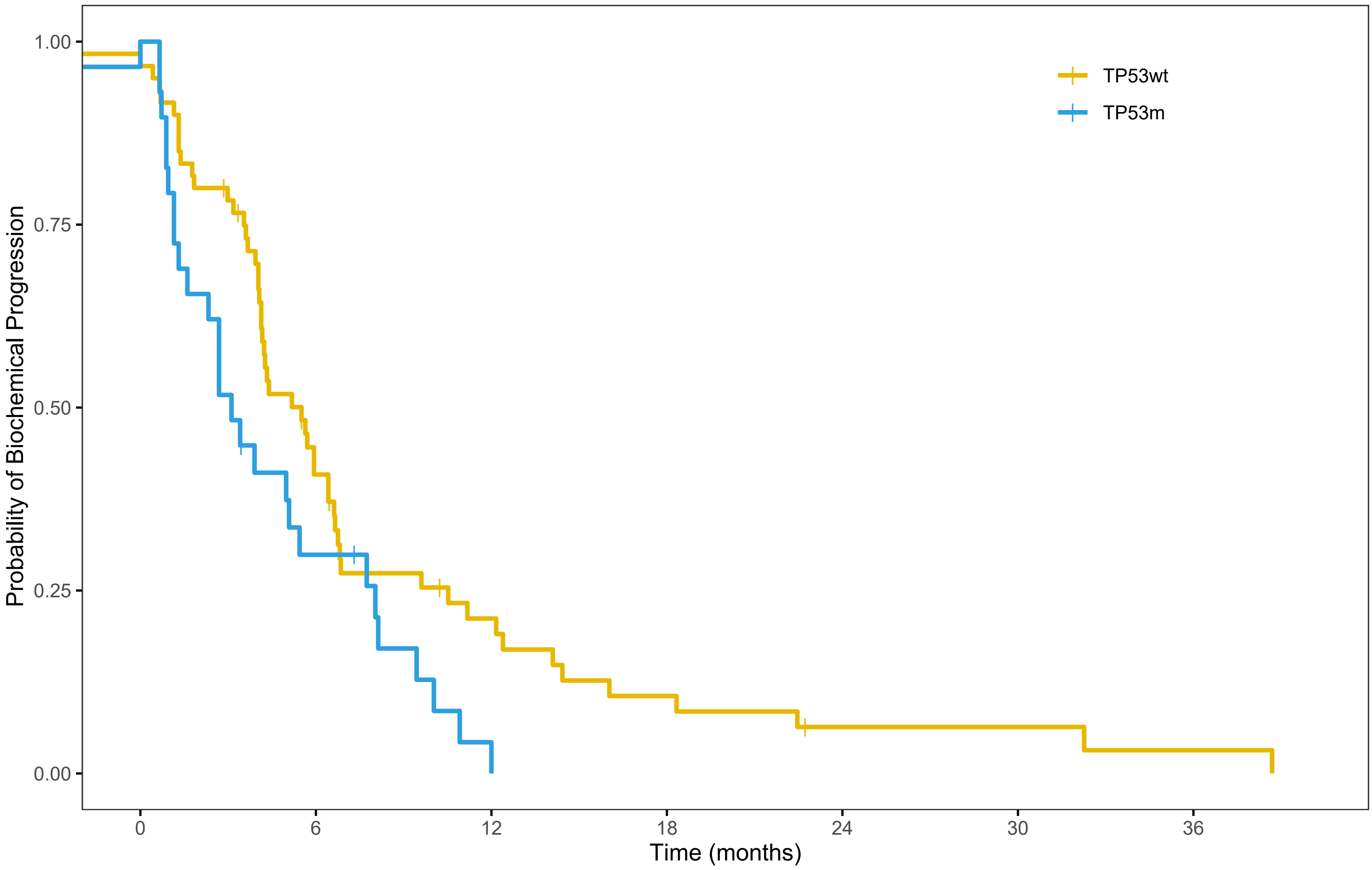

Kaplan-Meijer curves for the biochemical progression-free survival probability per TP53-mutational status.

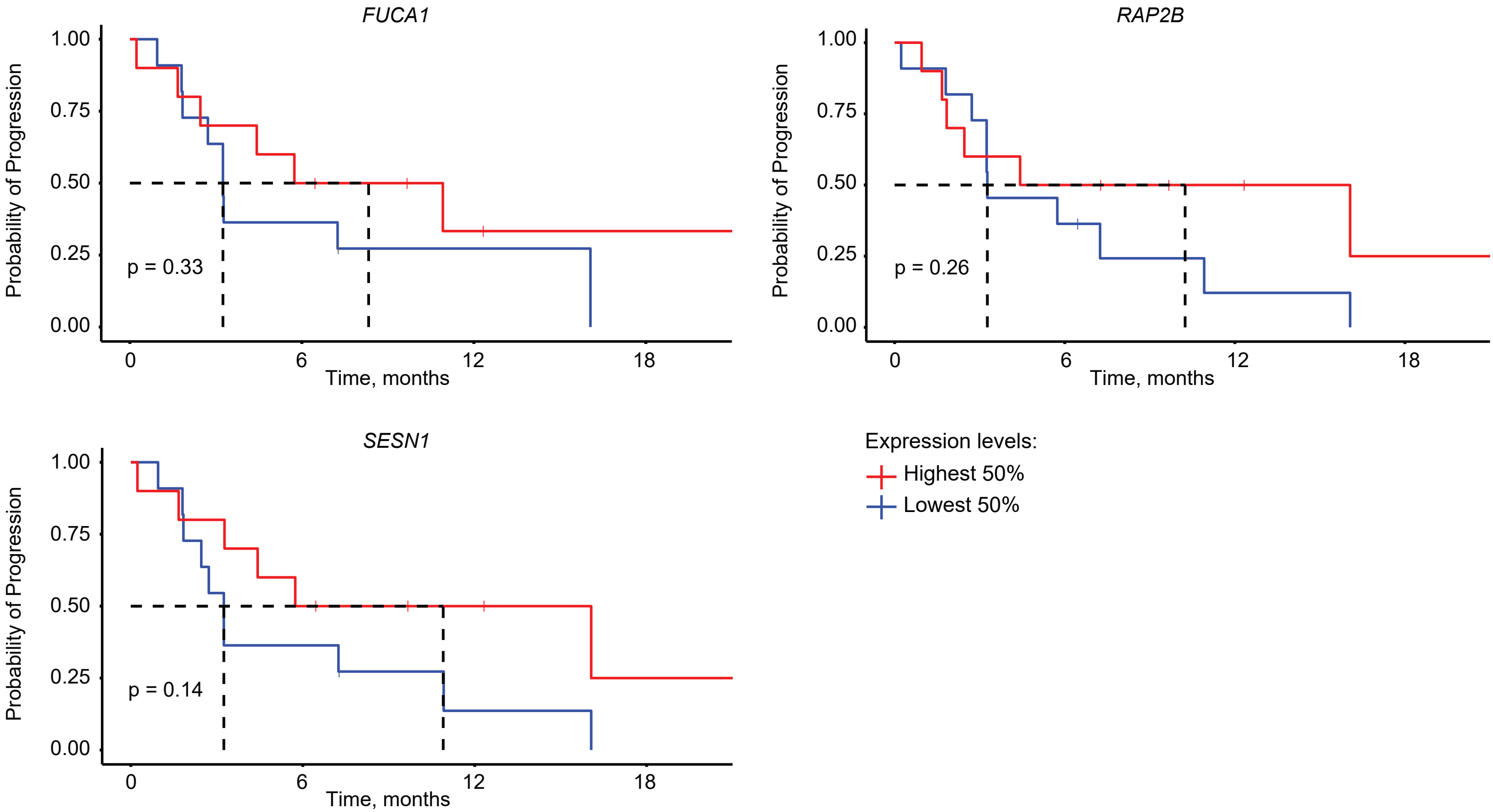

Kaplan-Meier curves for the progression-free survival based on the expression of FUCA1, RAP2B, and SESN1.
